# Can’t I continue to exercise here? Exploring experiences, barriers, and facilitators for physical therapists and survivors of cancer to promote exercise maintenance

**DOI:** 10.1007/s11764-025-01767-8

**Published:** 2025-05-28

**Authors:** S. C. Agasi-Idenburg, M. M. J. Joosten, M. Hoedjes, L. M. Buffart, C. S. Kampshoff, M. M. Stuiver

**Affiliations:** 1 Division of Psychosocial Research and Epidemiology, Netherlands Cancer Institute, Amsterdam, The Netherlands; 2 Research Group Innovation of Movement Care, University of Applied Sciences Utrecht, Utrecht, the Netherlands; 3 Faculty of Health, Amsterdam University of Applied Sciences, Amsterdam, The Netherlands; 4 Corps - Centre of Research on Psychological and Somatic Disorders, Department of Medical and Clinical Psychology, Tilburg University, Tilburg, The Netherlands; 5 Department of Medical BioSciences, Radboud University Medical Center, Nijmegen, The Netherlands

**Keywords:** Cancer, Exercise maintenance, Primary care, Qualitative research

## Abstract

**Purpose:**

Maintaining exercise behavior is crucial for cancer survivors, yet adherence to exercise recommendations remains low. This study explores the experiences and perspectives of community-working physical therapists and survivors of cancer regarding barriers and facilitators that support the maintenance of exercise behavior post-treatment.

**Methods:**

A qualitative, exploratory focus group design was employed, using purposive sampling to recruit oncology physical therapists and cancer survivors who had undergone physical therapy. The study assessed current physical therapy practices, barriers, and facilitators to exercise maintenance through thematic content analysis Braun and Clarke.

**Results:**

Six focus groups with 26 participants (12 cancer survivors and 14 physical therapists) revealed three main themes: (1) transition challenges from supervised therapy to independent exercise, (2) environmental constraints on exercise adherence, and (3) motivators and supportive factors to help independent exercise. Transition challenges included a lack of knowledge and skills, persistent symptoms, and psychological recovery. Environmental constraints involved prioritizing exercise over work and family and limited financial resources. Motivators and supportive factors included goal-setting, gradual reduction in physical therapy sessions, and building confidence in self-management among cancer survivors.

**Conclusions:**

Both physical therapists and cancer survivors experience challenges in concluding the treatment relationship. The identified facilitators for independent exercise can assist physical therapists in developing effective exercise programs that promote patient independence during and after physical therapy treatment.

**Implications for Cancer Survivors:**

Understanding these barriers and facilitators can help tailor interventions that enhance long-term exercise adherence, ultimately improving health outcomes and quality of life for cancer survivors.

**Supplementary Information:**

The online version contains supplementary material available at 10.1007/s11764-025-01767-8.

## Introduction

It is well-established that exercise interventions during and after cancer treatment reduce symptom load and improve physical function and quality of life [1–3]. In the long-term, sustained exercise behavior is associated with improved patient outcomes, including, for some cancers, better cancer-specific and overall survival [4, 5]. While the positive effects of exercise training for cancer survivors can persist for months following the conclusion of controlled research studies, there is a significant gap in knowledge regarding the translation of these findings into physical therapy-led [6] community-based exercise programs. To effectively support the increasing number of survivors, it is crucial to gain a deeper understanding of how these programs can be maintained in practice beyond the duration of short-term research projects or grant funding [7].

One of the studies that evaluated the long-term effectiveness of high-intensity versus low-to-moderate intensity exercise on physical activity and physical fitness in survivors of cancer is the randomized controlled Resistance and Endurance Exercise After ChemoTherapy (REACT) study [6, 8, 9]. In that study, a group of 278 patients with diverse cancer types treated with adjuvant chemotherapy took part in either a moderate-intensity or high-intensity, 12-week resistance, and endurance exercise intervention and were followed up to 64 weeks (long-term) after primary cancer treatment. At the long-term point, there was no difference in objectively measured physical activity between the groups. In both groups, intervention-induced improvements in cardiorespiratory fitness (VO2-peak, determined by a cardio-pulmonary exercise test) were maintained at long-term follow-up. However, overall fitness remained poor compared to healthy individuals, with an average range in Peak Vo2 of 22–25 (ml/kg/min). No discernible difference in physical activity, assessed by accelerometers, between the HI and MLI groups was found [10].

Other research also indicates that the effects of exercise interventions, whether supervised exercise or unsupervised physical activity programs have limited long-term benefits among cancer survivors. This includes compliance with physical activity recommendations, improvements in VO2 peak, muscle strength, and overall quality of life [11–14]. This can be explained, in part, by the non-maintenance of exercise behavior of survivors [11, 15, 16]. Indeed, cancer survivors are, on average, less physically active compared to age-matched peers who have not had cancer [11, 16, 17].

Exercise interventions are unlikely to elicit sustained physical activity or exercise behavior change unless they incorporate targeted behavioral change techniques [14, 18]. A systematic review and meta-analysis that studied the maintenance of physical activity behavior change in survivors of cancer provided an overview of used behavioral change techniques in 27 randomized controlled trials to support exercise maintenance of cancer survivors [14]. Ineffective interventions were less likely to include the behavioral change techniques (BCTs) “action planning,” “graded tasks,” and “social support (unspecified).” The effective use of these behavioral change techniques requires additional knowledge and skills of exercise specialists delivering exercise interventions to cancer survivors.

In the Netherlands, supervised exercise during and after cancer treatment is typically provided by physical therapists in primary care settings. Physical therapists who work with cancer survivors often pursue additional postgraduate education in oncology. This advanced training can be obtained through various shorter courses or one of the two available Master’s level programs in oncology. The focus of these courses is on advanced exercise physiology, understanding the effects of cancer treatment, and adapting exercise prescriptions to address disease and treatment-related side effects and risks. Although behavioral change techniques are also covered in this postgraduate education in oncology, these are not the main focus.

To improve practice, it is first necessary to better understand to what extent physical therapists working in oncology explicitly consider the maintenance of exercise behavior when delivering exercise interventions. Additionally, gaining insights into their perceived competence, barriers, beliefs, and values regarding supporting exercise behavior maintenance in patients is crucial. Likewise, to improve patient-therapist interaction, it is vital to understand the experiences survivors of cancer had with their physical therapist. This includes how the physical therapist guided them through their exercise program and toward sustainable self-managed exercise behavior. Furthermore, it is important to assess how well patients felt equipped for self-management after the exercise intervention ended.

Therefore, our research question was: To what extent do physical therapists working in oncology consider and support the maintenance of independent exercise behavior in survivors of cancer, and what are the experiences and perspectives of both therapists and patients regarding barriers and facilitators to maintaining exercise independently? As a first step to improving sustainable exercise behavior after physical therapy for cancer survivors, we conducted a qualitative study exploring these aspects.

## Methods

### Study design

This study is part of the larger research project, which aimed to identify the optimal content, design, and usability of a practical toolkit for physical therapists to support exercise maintenance among cancer survivors, through qualitative research among users (current study), desk research, and co-creation design. The current study employed a qualitative approach, specifically phenomenology, within a constructivist research paradigm, to deeply understand the experiences and perspectives of participants. The constructivist research paradigm is based on the idea that reality is constructed through human experiences and interactions. It posits that knowledge is subjective and shaped by the context in which it is developed. In this paradigm, researchers aim to understand the meanings and interpretations that individuals or groups assign to their experiences. By employing a qualitative approach, specifically phenomenology, within a constructivist framework, this study seeks to deeply explore and understand the lived experiences and perspectives of participants.

### Participants

A purposeful sampling strategy was employed to select participants meeting specific criteria relevant to the research objectives. Eligible physical therapists were educated in oncology and currently treating cancer survivors with cancer-related symptoms in primary care. Inclusion criteria for participants in the focus groups for survivors of cancer included any current or past experiences with a physical therapist-led exercise program in primary care for the treatment of cancer or related symptoms, regardless of the time elapsed since their last oncologic treatment. The present study utilized a maximum variation sampling strategy to promote heterogeneity among participants in the patient focus groups. Specifically, cancer survivors were recruited to participate in the focus group discussions, representing a diverse range of cancer types and physical activity preferences (i.e., engaging in sports or not). This sampling approach was employed to capture a wide spectrum of perspectives and experiences within the target population.

### Recruitment

Physical therapists were recruited through a multi-pronged recruitment strategy. Physical therapists who have successfully completed post-graduate oncology education can register with a national network or one of the regional networks of physical therapists with added oncology qualifications. For the current study, we aimed to recruit physical therapists with post-graduate training in oncology of any level. Therefore we first recruited three physical therapists who were members of the national or a regional (southern Netherlands) network. These physical therapists were subsequently asked to share recruitment calls within their respective regional networks. This approach aimed to leverage the existing connections and communication channels within the specialized oncology physical therapy community to facilitate the identification and enrollment of eligible participants for the study. Additionally, we employed snowball sampling to expand our participant pool. Snowball sampling, also known as chain-referral sampling, is a non-probability sampling method where existing study participants recruit future participants from their network [19]. Physical therapists who had indicated their willingness to participate in the focus groups were asked to suggest other colleagues who might be interested in participating in the focus groups, who were then subsequently approached.

To recruit participants for the focus groups of cancer survivors, initially, the physical therapists who had participated in the focus groups were asked to identify and inform eligible cancer survivors. Additionally, we asked physical therapists registered in the national network to identify eligible cancer survivors and ask them whether they would be interested in participating in the focus groups. We also announced the study in the monthly newsletter of “Tegenkracht,” an organization with the goal of providing sports guidance to people diagnosed with cancer. Interested cancer survivors received the study information and were included by the study team if they provided informed consent.

All participants, physical therapists and survivors of cancer received both written and verbal information about the (overall) study purpose, the development of a toolkit, and the goals for the current study to elicit their views and experiences with regard to facilitating factors and limiting factors to help patients in physical therapy sessions to initiate exercise behavior that can be maintained after physical therapy sessions end. Focus groups were planned at least three days after information was given to provide potential participants ample time to make a definite decision on participating in the focus groups. Informed consent, also for the collection of audiotaped data, was obtained from each participant before data collection. Collected data were stripped of information that could potentially pose a threat to the anonymity of participants. In data transcripts and analysis, participants were given a pseudonym. Ethical approval was granted by the Institutional Review Board of the Netherlands Cancer Institute, reference number: IRBd20-356.

### Data collection

A team of five experienced qualitative researchers with different backgrounds (i.e., psychology, physical therapy, human movement sciences, and clinical health sciences) conducted the data collection. Due to the COVID-19 pandemic, focus groups were conducted online, as has been done in other studies during this period [20–22]. Recruitment concluded after evaluating the richness of data from previous focus groups, the exploratory nature of the research question, the data collection method, and the diversity of respondents. Financial constraints and the need to limit participant numbers were also considered. Including additional participants to gain more depth in the results was weighed against the potential burden on more participants, with the understanding that it would yield limited extra variation in the results. All focus groups were led by one researcher who primarily asked the questions (the moderator), and one researcher who, in addition to taking notes on notable observations, occasionally posed supplemental questions (the observer).

Interview guides for the physical therapist groups were initially developed by the research team, based on the literature concerned with barriers to and facilitators of exercise maintenance for cancer survivors with a diverse range of cancer types [23–25], and subsequently discussed and refined with input from the three oncology physical therapists. The interview guide used in the focus group with the physical therapist can be found in Appendix 1. The interview guide for the focus groups with survivors of cancer was developed based on literature [23–25] and the results of the focus groups with physical therapists. It facilitated discussions on the physical therapy received (both general and specifically aimed at promoting independent exercise maintenance), as well as the barriers and facilitators to maintaining independent exercise. Refer to Appendix 2 for the interview guide used in the focus group with cancer survivors. Small adjustments were made to the interview guide, based on preceding focus group discussions. This allowed for the inclusion of topics that emerged in the earlier focus groups and appeared pertinent for addressing the research question.

Focus groups were conducted via an online videoconferencing platform, Microsoft Teams, to provide a comfortable and confidential environment. Before starting the focus group discussion, the moderator provided a short overview of the research project and informed the participants about their rights to prematurely terminate their participation in the study without negative consequences. After all participants had briefly introduced themselves, they were first asked to elaborate on their exercise preferences. In the physical therapy focus groups, questions were posed to the group about their experiences with and views of current physiotherapy practices in guiding cancer survivors to maintain exercising after ending physiotherapy treatment and barriers and facilitators of exercise maintenance. In the focus groups with cancer survivors, questions were posed to the group about their experiences with the transition from physical therapy-guided training to exercising on their own. The (lack of) guidance cancer survivors experienced in this transition, and barriers and facilitators making unsupervised sustainable exercise behavior possible. All focus groups lasted approximately 90 min. The sessions were audio-recorded and transcribed verbatim. Field notes were compiled, containing observations regarding notable occurrences within the focus group, as well as methodological notes and suggestions for subsequent focus group sessions.

### Data analysis

The steps taken in the data analysis were discussed within the larger research group of authors involved in this study. Two researchers (S.C.A-I and M.M.J.J) conducted the data analysis using a reflective thematic analysis approach [26] according to the method of Braun en Clarke [27, 28]. Reflective thematic analysis entailed the initial identification of text segments that contained information relevant to addressing the research question. Theses text segments were given an inductive code, using latent coding, based on the narrative extracted from the segments. As researchers, we adopt a constructivist epistemological position, which acknowledges that knowledge is constructed through social processes and interactions. This perspective guided our approach to data analysis, emphasizing the co-construction of meaning between the researchers and the data. By engaging in reflexive practices and collaborative discussions, we aimed to ensure that our interpretations were grounded in participants’ experiences and the context of the study. One researcher, a 27-year-old professional, had a background in nursing and clinical health sciences, while the other, a 56-year-old experienced academic, had expertise in clinical health sciences and physical therapy. A transcript of one focus group was coded independently by both researchers and coding was discussed afterwards. This initial coding approach between the researchers differed in the level of detail. However, the essence of the assigned codes was consistent between the two researchers. Subsequently, one of the two researchers coded the remaining focus groups. Next, both researchers discussed the organization of the codes in overarching categories [29]. These categories consisted of codes with similar content or shared meaning. This phase of the data analysis was aided by the use of Atlas ti v. 22 Scientific Software Development GmbH Berlin, Germany. The final phase of data analysis, the process of identifying themes, was facilitated by the use of Visio software, Microsoft, Redmond USA. In this phase, a code tree was created in Microsoft Visio, displaying both the codes and categories. Subsequently, in consensus with both researchers, themes were inductively identified. To validate the proposed themes, a review was conducted by referring back to the narratives of the participants, ensuring that the themes exhibited sufficient coherence. Minor adjustments were made based on this assessment, leading to the identification of the final themes. In our analysis, we examined the data from patients’ and physical therapists’ focus groups separately. It became evident that there were overlapping themes and shared perspectives across both groups. Recognizing the value in integrating these insights, we proceeded to merge the results. This approach allowed us to create a more holistic understanding of the factors influencing exercise maintenance after supervised training.

Researchers engaged in reflexivity, acknowledging their own perspectives and potential biases, and actively participated in peer debriefing sessions where colleagues provided feedback and challenged assumptions. This collaborative approach facilitated the identification and mitigation of any unconscious biases [29]. Furthermore, regular team meetings were conducted to deliberate on and scrutinize findings.

## Results

Focus groups with physical therapists were held between March and April 2021. Subsequently, focus groups with cancer survivors were conducted between March and April 2022. In total, 27 participants were recruited, comprising 14 physical therapists and 13 survivors of cancer. Sociodemographic data for participants by group are summarized in Table 1.

**Table 1:** Participants’ characteristics

Physical therapists			
Participant number	F/M	Education	Work setting
Th1	F	Oncology-trained lymphedema physical therapist, second year Master Oncology	(Community) primary care setting
Th2	F	Master oncology, oncology-trained lymphedema physical therapist, biomedical scientist,	(Community) primary care setting
Th3	F	Master oncology, physical therapist	(Community) primary care medical center
Th4	F	Physical therapist in second year of master oncology	(Community) primary care setting
Th5	F	Master oncology trained lymphedema physical therapist, practice owner	(Community) primary care setting
Th6	F	Oncological-trained lymphedema physical therapist	(Community) primary care setting
Th7	F	Oncological-trained lymphedema physical therapist	(Community) primary care setting
Th8	F	Oncological-trained physical therapist	(Community) primary care setting
Th9	F	Oncological-trained physical therapist	(Community) primary care setting
Th10	F	Oncological-trained lymphedema physical therapist	(Community) primary care setting
Th11	M	Oncological-trained lymphedema physical therapist	(Community) primary care setting
Th12	F	Oncological-trained lymphedema physical therapist	(Community) primary care setting
Th13	F	Oncological-trained lymphedema physical therapist, practice owner	(Community) primary care setting and hospital
Th14	M	Master Oncology, lymphedema physical therapist	(Community) primary care setting
Cancer survivors			
Participant number	F/M	Background	Disease
S1	M	Manager, medical fitness	Waldenstrom’s disease
S2	F	Stay home mom, not physically active	Breast cancer
S3	F	Stay home mom, several months post-surgery, physical therapy treatment	Breast cancer
S4	F	Stay home mom, sport triathlon, running, trains with cancer trainings club	Breast cancer
S5	F	Discontinued education aimed at becoming a fitness trainer due to illness, sport	Breast cancer
S6	F	Office worker, cycles to work, walks with dogs	Lung cancer
S7	F	Office worker, few months after neoadjuvant chemotherapy, medical fitness, active bootcamp	Breast cancer
S8	F	Loves sports but stopped, due to cancer, physical therapy	Breast cancer
S9	F	Teacher, walks	Breast cancer
S10	M	Office worker, walks long distances	Lymphoma
S11	M	Executive director physical fitness, not active	Colorectal cancer
S12	F	Industry planning manager, Triathlon, sport	Breast cancer

*Th* physical therapist, *S* survivor of cancer, *F* female, *M* male

**Fig. 1:**
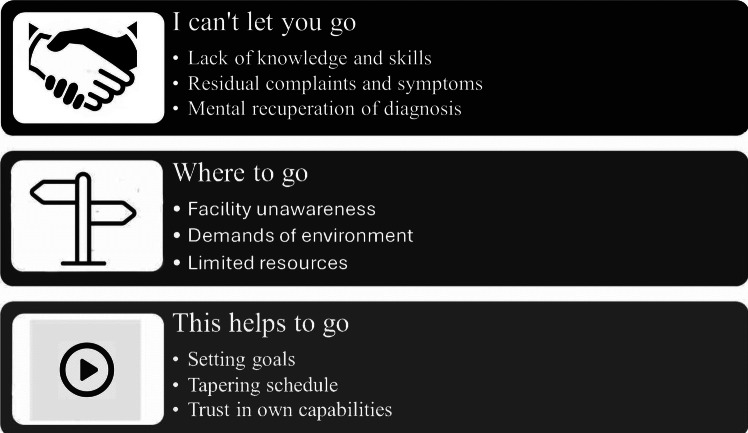
Themes

We identified three overarching themes in relation to exercise maintenance for cancer survivors after supervised training, see Fig. 1. These include (1) “I can’t let you go,” which concerned challenges to end the physical therapist-patient treatment relationship; (2) “Where to go?” which included facility unawareness, limited resources and demands of the environment; and (3) “This helps to go,” which related to motivators and supportive factors for ending the physical therapist- patient treatment relationship positively.

We provide a detailed description of each of these themes, along with reference to specific illustrative quotes in Table 2. Quotes were selected based on their clarity in presenting interesting results that contribute to understanding the research question. Quotes are numbered, with the numbers corresponding to the numbering of the quotes in Table 2. Following the quote number, the participant number is indicated. Physical therapists are denoted by the abbreviation Th, for therapist. Cancer survivors are indicated by the letter S for survivors.

**Table 2:**
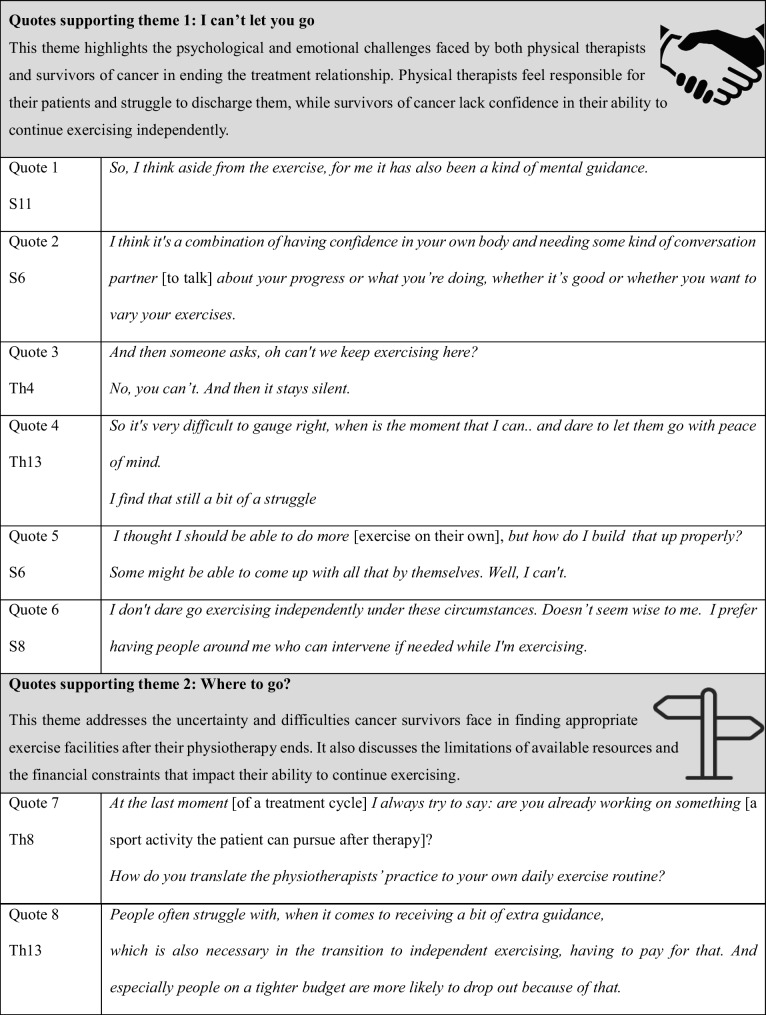

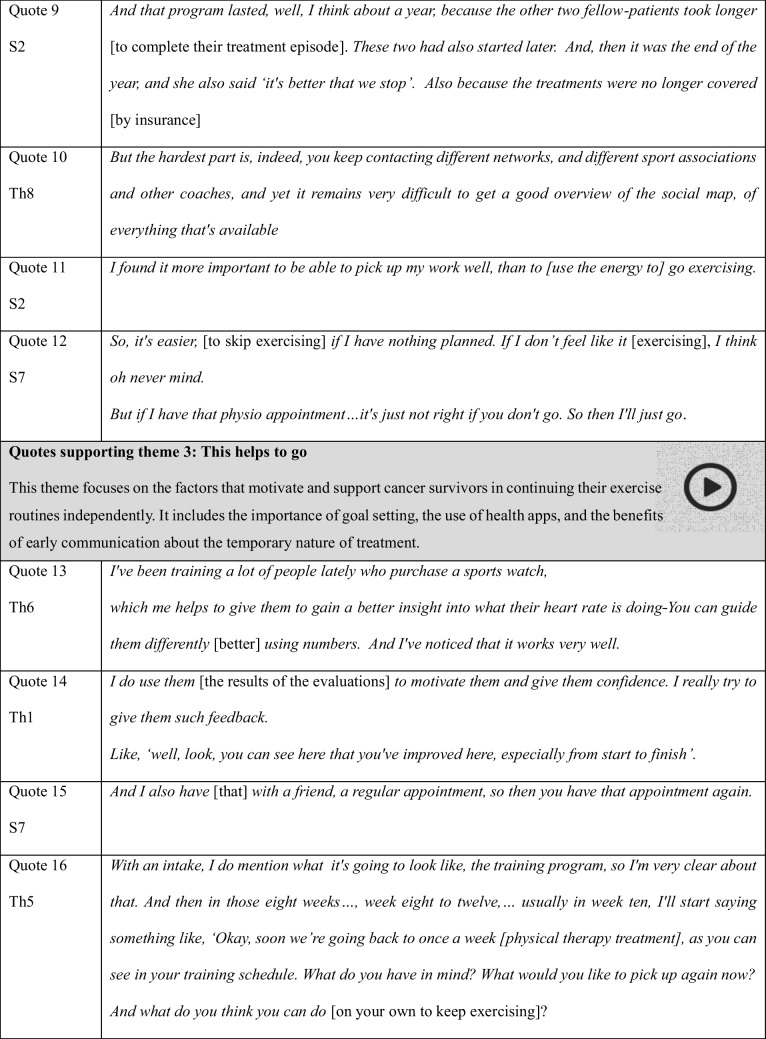
Explanation of themes and illustrative quotes highlighting key themes and participant contributions

Physical therapists are denoted by the abbreviation Th, for therapist. Survivors of cancer are indicated by the letter S for survivors

### Theme 1: I can’t let you go

Both physical therapists and cancer survivors highlighted the psychological repercussions of the diagnosis, precipitating a phase of mental recuperation during which cancer survivors lacked the initiative to proactively manage their recovery (Quote 1, S11). Several cancer survivors indicated that after a period of uncertainty, it is very comforting to have the opportunity to discuss various matters with the physical therapist (Quote 2, S6). Physical therapists aim to provide guidance during this period.

However, after this initial period, physical therapists find it challenging to discharge cancer survivors. They feel responsible and want to support their patient in many ways. They articulated finding it difficult to ask cancer survivors to take responsibility for their own exercise training (Quote 3, Th4). For some physical therapists, this was because they had trouble discerning the readiness of a patient to initiate independent exercise regimens (Quote 4, Th13). Several other physical therapists indicated a lack of competence in encouraging cancer survivors, who have no prior engagement in sporting activities (such as running, fitness, or hockey), to take up independent physical activities. These physical activities include daily life tasks such as walking to buy groceries, cycling to work, or being active in the garden. Also, some physical therapist preferred “their” cancer survivors to continue training under the care of other professionals, like medical fitness trainers instead of giving them the responsibility of training on their own.

Both cancer survivors and physical therapists questioned whether cancer survivors possess sufficient skills and knowledge to independently engage in exercise training. One physical therapist expressed the concern that cancer survivors will return with injuries when they exercise without guidance. Cancer survivors experienced feelings of insecurity and lacked confidence in their knowledge and abilities to independently engage in exercise training as well (Quote 5, S6). Despite cancer survivors feeling incapable of continuing training independently, this did not result in cancer survivors requesting physical therapists to teach them these skills. Also, they harbored uncertainties regarding their bodies or contemplated the possibility of cancer recurrence when experiencing unfamiliar sensations (Quote 6, S8).

Furthermore, cancer survivors, pondering the effects of cancer treatments, voiced uncertainties about their capacity to exert physical strain on their bodies. Some cancer survivors articulated a desire to remain exercising under the guidance of a physical therapist, expressing an incomplete recovery status. Finally, several physical therapists noted that cancer survivors can be reluctant to cease physiotherapy-guided training, due to unrealistic expectations regarding their recovery capabilities.

### Theme 2: where to go?

During physiotherapy treatment, not all cancer survivors, or even all physical therapists, seemed to fully appreciate the temporary arrangement of the therapeutic relationship. Some physical therapists reported to end their treatment only when reimbursement ends. Other physical therapists said they do prepare the patient for the upcoming end of treatment, and, in that context, ask cancer survivors where they would want to continue their exercise training (Quote 7, Th8). Some cancer survivors found it difficult to know where to go, and physical therapists often felt unequipped to help them find their way, within the constraints of available services and the cancer survivors’ available budget (Quote 8, Th13).

The physical therapists were most inclined to send cancer survivors to medical fitness clinics or back to their former sport. One patient mentioned he continued exercising in a medical fitness group, merely because other cancer survivors in that group were not ready to leave yet and he wanted to support his fellow exercisers (Quote 9, S2).

Some physical therapists said they find it difficult to know where to refer after physical therapy treatment because there is not a lot of expertise about oncology in regular sports facilities (Quote 10, S8).

Also, both the cancer survivors and the physical therapists recognized that regular fitness facilities can be too expensive for the limited financial resources of some cancer survivors. In that context, one physical therapist mentioned the possibility of municipal subsidies but concurrently indicated that such opportunities varied so extensively across municipalities that he had completely lost track of the different options. Both physical therapists and cancer survivors identified social obligations, particularly work-related commitments, as significant impediments to independently undertaking physical training (Quote 11, S2).

Cancer survivors explained that it is easier to justify (to themselves or others) allocating time to exercise when this is contextualized as receiving health care. When the structured support of scheduled physical therapy appointments ends, cancer survivors often find it challenging to independently prioritize time for regular physical exercise. Additionally, several cancer survivors cited a lack of motivation, resulting in the postponement or cancelation of training sessions when not scheduled with the physical therapist (Quote 12, S7).

### Theme 3: this helps to go

Key factors for exercising independently from a physical therapist, identified by both groups, included goal-setting, information on the importance of exercise, cancer survivors’ perception of their ability to contribute to their health, and insight into the outcomes of their physical exercise training. In this context, some physical therapists and cancer survivors highlighted the benefits of health apps and smartwatches (Quote 13, Th 6). Additionally, experiencing the positive effects of physical exercise training was mentioned as a motivational factor (Quote 14, Th1). Particularly cancer survivors emphasized the importance of regular appointments for exercise training within a fixed group within or outside healthcare facilities for motivation (Quote 15, S7). One physical therapist, who also worked in a rehabilitation setting, stressed the significance of early communication with cancer survivors about the temporary nature of treatment to foster appropriate patient expectations (Quote 16, Th5). Another physical therapist emphasized the importance of cancer survivors engaging in independent physical training tailored to their individual training goals, both during and after physiotherapy treatment. The use of a tapering schedule, where contact moments with the physical therapist are progressively spaced over time, was also perceived as beneficial.

## Discussion

This study investigated the perspectives of physical therapists with postgraduate oncology training and survivors of cancer regarding sustainable exercise behavior following physical therapist-supervised exercise interventions. Our results show that both patients and physical therapists in the focus groups experienced challenges in transitioning from guided sessions to independent exercise maintenance. Reasons included survivors’ lack of knowledge on how to exercise independently and lack of initiative for autonomous exercise engagement. Survivors expressed a desire for continued supervision due to perceived limitations, with lingering discomfort and symptoms reinforcing this preference. Uncertainty about post-therapy options was noted by both groups, with financial barriers and limited alternative locations to continue exercising reported as a barrier by survivors. Additionally, survivors struggled to prioritize exercise amidst work and childcare responsibilities. Competing responsibilities were previously also reported as an important barrier to physical activity in adults with breast cancer receiving adjuvant treatment [30].

An important facilitator of autonomous exercise, as identified in our study, is the establishment of clear goals at the initiation of therapy. This was also recognized in previous research [31, 32]. In addition to goal setting, systematic reviews evaluating strategies to achieve sustainable physical activity behavior change in individuals with and beyond cancer highlight the importance of social support and action planning [14, 33]. Interestingly, the utilization of action planning was not reported in either the focus groups with physical therapists or with cancer survivors. This suggests that physical therapists may be insufficiently aware of the benefits of action planning in the treatment of patients. In a systematic review that reviewed behavior change techniques, physical therapists use when promoting physical activity in experimental and observational studies only seven different behavior change techniques were used in observational studies [34]. Action planning was ranked fourth among the seven behavior-change techniques that were reported in the three observational studies and was used in all nine experimental studies described [34].

Some of our findings were also reported in non-cancer populations [35–39], while others seem relatively cancer-specific [40–42]. For example, similar mechanisms relating to exercise maintenance seem to be at play for patients with low back pain [43] In that study, patients’ insufficient skills and confidence levels were major barriers and attributed to the need for patients to acquire new skills tailored to their (health) circumstances. Time constraints and scheduling conflicts were cited as significant barriers, particularly among patients new to home exercise routines. In our study, even survivors of cancer with prior exercise exposure encountered challenges in incorporating regular exercise into their routines, attributing difficulties to lingering symptoms and side effects of cancer and its treatments.

“Accountability” is a facilitator that is well-recognized in previous research. A recent qualitative study on exercise maintenance after multidisciplinary cancer rehabilitation identified “accountability” as an important facilitator [31]. The benefits of scheduling fixed exercise appointments to ensure consistent participation were previously also described in research among breast cancer survivors involved in group exercise programs or home-based training [30, 44]. Similar feelings were voiced by participants in our study. While having appointments with a physical therapist provides a sense of accountability, both cancer survivors and physical therapists in our study thought such feelings could also be achieved through scheduling exercise sessions with friends or peers of similar abilities. However, patients should be actively encouraged to organize such accountability within their own environment before physical therapy treatment ends.

In our study, most survivors of cancer and physical therapists acknowledged the advantages of exercising in groups. Schmidt et al. [45] also underscored the importance of fostering social connections as prostate survivors of cancer shift from supervised hospital-based exercise groups to independent community-based exercise. Participants who exercised alone or with their spouses post-program noted the lack of physical activity and social engagement with peers as notable absences [45].

While many of our findings echo previous research, our detailed analysis of the specific barriers and facilitators to exercise maintenance from both the perspectives of cancer survivors and physical therapists provides additional insights. This dual perspective approach offers a more comprehensive understanding of the challenges faced during the transition from supervised to independent exercise. Unlike previous studies that primarily focused on patient perspectives, our research highlights the critical role of physical therapists in facilitating exercise maintenance and the challenges they experience. The identification of physical therapists’ lack of awareness regarding cognitive behavioral techniques, such as action planning, is a novel finding that underscores the need for targeted educational interventions for physical therapists [31].

We recruited survivors of cancer for our study via an organization aimed at offering sports guidance for survivors of cancer. This organization provides exercise testing and helps people living with or beyond cancer to find appropriate exercise support tailored to their needs and abilities. Therefore, we included the individuals whom physical therapists are likely to encounter in clinical practice, those who are interested in exercise but struggling to sustain independent exercise behavior. To avoid the overrepresentation of exercise-interested survivors of cancer, we enlisted physical therapists from our network to invite survivors of cancer lacking interest in exercise to participate in the study.

Consistent with our constructivist epistemological stance, the data analysis was conducted by two researchers with diverse professional backgrounds. This diversity enriched the data interpretation, facilitating a deeper understanding of the findings. By discussing different perspectives and interpretations during the analysis, we ensured that the findings were not solely dependent on a single viewpoint, thereby reducing the chance of potential biases or blind spots. By integrating multiple perspectives, the validity and “trustworthiness” of the research are strengthened as the conclusions are more likely to be comprehensive and reflective of the data [46]. This collaborative approach underscores our commitment to co-constructing knowledge through social processes and interactions, ensuring that our findings are grounded in the varied experiences and contexts of the study participants. Although actively engaging participants in member checking would have further ensured that our findings were grounded in their varied experiences and contexts, we chose not to do so to limit the burden on participants.

However, a limitation of this study is the predominance of female participants, both within the physical therapist group and the survivors of cancer group. We do feel that this overrepresentation of women in physical therapists is explainable. Many post-academic oncologically trained physical therapists are lymphedema therapists. Although data on the percentage of female lymphedema therapists in the Netherlands is lacking, an international study indicates a clear overrepresentation of women in this group [47]. In the participant group of survivors of cancer, the majority were women diagnosed with breast cancer. This gender imbalance, in both group participants, may have influenced the findings, as the experiences and challenges faced by male survivors of cancer or those with different types of cancer might not be fully represented. This overrepresentation of women could potentially skew the insights and themes identified, making it important to consider these limitations when interpreting the results. Future research should aim to include a more diverse participant pool to capture a wider range of perspectives and experiences.

Another hindrance in the study is the limited differentiation between physical activity and exercise behavior. Although the patient participants all underwent exercise training with a physical therapist and clearly spoke more from a perspective of exercise behavior, the concepts of physical activity and exercise as a more specific form of physical activity sometimes became blurred during the focus groups. It is important to note that the exercise training consisted of individualized physical therapy treatments and detailed insight into how the training was conducted was not available for each of the participants. However, considering the available guidelines, it is safe to assume the exercise sessions included components of strength and aerobic exercise [3].

The findings of this study can inform the development of targeted interventions to improve unsupervised exercise maintenance in survivors of cancer and enhance the quality of physical therapy. In particular, our findings underscore the need for practical strategies and tools for physical therapists to support sustainable exercise behavior in survivors of cancer. Physical therapists should be aware that already in the early stages of physical therapy treatment, emphasis should be placed on empowering the patient to engage in physical activity independently. Cognitive behavioral techniques such as goal setting and action planning can make a valuable contribution in this regard. Empowering survivors through education and self-management strategies is also essential. Providing resources such as informational booklets, and digital tools can help survivors integrate exercise into their daily routines despite their busy schedules.

## Conclusions

Both physical therapists and survivors of cancer in our focus groups indicated difficulties in (supporting) the transition from treatment in the physical therapy practice to independent physical activity. The primary facilitators identified to aid in this transition involve goal setting and gradually tapering off treatment, thus increasing patient responsibility. The significance of explicitly incorporating cognitive behavioral strategies in physical therapist-supervised exercise interventions seems to be insufficiently recognized by physical therapists. Obtaining such knowledge and skills would be a useful expansion of physical therapists’ toolkit and should be a priority for oncologic physical therapy education. Regular monitoring and evaluation of these interventions are essential to ensure their effectiveness.

## Supplementary Information

Below is the link to the electronic supplementary material.

Supplementary file1 (DOCX 24 KB)

Supplementary file2 (DOCX 24.9 KB)

## Data Availability

Yes, I have research data to declare. The data supporting the results of this manuscript were collected during the study. However, due to privacy concerns and the terms outlined in the consent forms, the data are only accessible to the researchers involved in this study. Sharing the data with external parties was not covered in the participants' consent, and therefore, the data cannot be made publicly available.
