# Supplementary material for: Can’t I continue to exercise here? Exploring experiences, barriers, and facilitators for physical therapists and survivors of cancer to promote exercise maintenance
Source: J Cancer Surviv. 2025 May 28;20(4):1684–96. doi: 10.1007/s11764-025-01767-8 (PMC13375871; doi:10.1007/s11764-025-01767-8)
Supplement: Supplementary file 1 — Supplementary file1 (DOCX 24 KB) [file 11764_2025_1767_MOESM1_ESM.docx]

Appendix 1. INTERVIEW GUIDE FOCUS GROUP PHYSICAL THERAPISTS

| INTRODUCTION QUESTIONS | |
| --- | --- |
| Primary question | *When you look at your current approach in practice when guiding oncology patients to the phase of independent training, what aspects are you satisfied with, in terms of approach and outcomes?* |
| KEY QUESTION 1 OWN EXEPERIENCE | |
| Primary question | *You guide oncology patients in your practice. Can you briefly explain your approach to guiding patients in the transition from training in your physiotherapy practice to independent training?* |
| Sub questions | *Can you name some examples of materials/facilities, exercises, and/or techniques you use to facilitate the transition from guided training to independent training?* |
| KEY QUESTION 2 IMPEDING FACTORS | |
| Primary question | *We also aim to gain insight into the factors that hinder the transition from training in the physical therapy practice to independent training.*  *What do you consider the most important factors that hinder the transition to independent training?* |
| Sub questions | Depending on the answer, the moderator asked follow-up questions such as:   - *Can you think of environmental factors, such as lack of materials or techniques?* - *Can you think of behavior-related factors such as lack of motivation in the patient?* |
| KEY QUESTION 3 PROMOTING FACTORS | |
| Primary question | *What do you consider the most important factors that promote the transition from training in the physical therapy practice to independent training?* |
| Sub questions | *What materials/facilities, exercises, and /or techniques do you use to leverage these factors?* |
| KEY QUESTION 4 REQUIREMENTS | |
| Primary question | *What do you need as a physical therapist to guide oncology patients to the phase of independent training?* |
| Sub questions | - *What skills/competencies do you think of?* - *What kind of materials/facilities do you think of?* |
| GOODBYE | |
| Primary question | *Is there anything else you say is important to discuss, in terms of sustaining exercise after physical therapist’s guidance ended?* |
