# Supplementary material for: Can’t I continue to exercise here? Exploring experiences, barriers, and facilitators for physical therapists and survivors of cancer to promote exercise maintenance
Source: J Cancer Surviv. 2025 May 28;20(4):1684–96. doi: 10.1007/s11764-025-01767-8 (PMC13375871; doi:10.1007/s11764-025-01767-8)
Supplement: Supplementary file 2 — Supplementary file2 (DOCX 24.9 KB) [file 11764_2025_1767_MOESM2_ESM.docx]

Appendix 2. INTERVIEW GUIDE FOCUS GROUP SURVIVORS OF CANCER

| INTRODUCTION QUESTIONS | |
| --- | --- |
| Primary question | - *Can you introduce yourself?* - *Can you explain what exercise and sports meant to you before the cancer diagnosis?* |
| KEY QUESTION 1 OWN EXPERIENCE | |
| Primary question | - *Some people find it difficult to keep exercising after supervised workouts with the physical therapist have stopped.* - *How was it for you?* |
| Sub questions | Depending on the answer, the moderator asked follow-up questions about:   - Personal factors - Physiotherapy-related factors, - Contextual factors, - Environmental factors such as training facilities or money. |
| KEY QUESTION 2 IMPEDING FACTORS | |
| Primary question | *Based on your own experiences, what factors do you think contribute to people with a cancer diagnosis stopping exercise after physical therapy ends?* |
| Sub questions | Depending on the answer, the moderator asked follow-up questions about:   - Personal factors such as behavior-related factors or motivation. - Physiotherapy related factors such as the extent to which the physical therapist prepared the patient for independent exercise - Social factors such as demands of family life or work responsibilities - Contextual factors, such as training facilities or financial constraints |
| KEY QUESTION 3 PROMOTING FACTORS | |
| Primary question | *Based on your own experiences, what factors do you think contribute to people with a cancer diagnosis continue exercise after physical therapy ends?* |
| Sub questions | Depending on the answer, the moderator asked follow-up questions about:   - Personal factors such as behavior-related factors or motivation. - Physiotherapy related factors such as the extent to which the physical therapist prepared the patient for independent exercise - Social factors such as support from family, friends or work relations - Contextual factors, such as training facilities |
| GOODBYE | |
| Primary question | *Is there anything else you say is important to discuss, in terms of sustaining exercise after physical therapist’s guidance ended?* |
